# Acetate and succinate benefit host muscle energetics as exercise‐associated post‐biotics

**DOI:** 10.14814/phy2.15848

**Published:** 2023-11-08

**Authors:** Ahmed Ismaeel, Taylor R. Valentino, Benjamin Burke, Jensen Goh, Tolulope P. Saliu, Fatmah Albathi, Allison Owen, John J. McCarthy, Yuan Wen

**Affiliations:** ^1^ Department of Physiology, College of Medicine University of Kentucky Lexington Kentucky USA; ^2^ Center for Muscle Biology University of Kentucky Lexington Kentucky USA; ^3^ Buck Institute for Research on Aging Novato California USA; ^4^ Department of Pharmacology and Nutritional Sciences, College of Medicine University of Kentucky Lexington Kentucky USA; ^5^ Department of Athletic Training College of Health Sciences University of Kentucky Lexington Kentucky USA; ^6^ Division of Biomedical Informatics, Department of Internal Medicine, College of Medicine University of Kentucky Lexington Kentucky USA

**Keywords:** exercise, metagenomics, microbiome, skeletal muscle

## Abstract

Recently, the gut microbiome has emerged as a potent modulator of exercise‐induced systemic adaptation and appears to be crucial for mediating some of the benefits of exercise. This study builds upon previous evidence establishing a gut microbiome‐skeletal muscle axis, identifying exercise‐induced changes in microbiome composition. Metagenomics sequencing of fecal samples from non‐exercise‐trained controls or exercise‐trained mice was conducted. Biodiversity indices indicated exercise training did not change alpha diversity. However, there were notable differences in beta‐diversity between trained and untrained microbiomes. Exercise significantly increased the level of the bacterial species *Muribaculaceae bacterium DSM 103720*. Computation simulation of bacterial growth was used to predict metabolites that accumulate under in silico culture of exercise‐responsive bacteria. We identified acetate and succinate as potential gut microbial metabolites that are produced by *Muribaculaceae bacterium*, which were then administered to mice during a period of mechanical overload‐induced muscle hypertrophy. Although no differences were observed for the overall muscle growth response to succinate or acetate administration during the first 5 days of mechanical overload‐induced hypertrophy, acetate and succinate increased skeletal muscle mitochondrial respiration. When given as post‐biotics, succinate or acetate treatment may improve oxidative metabolism during muscle hypertrophy.

## Graphical abstract

Metagenomic sequencing of fecal DNA after progressive weighted wheel running (PoWeR) was performed, identifying increased abundance of *Muribaculaceae bacterium*. Computational simulation of bacterial growth predicted acetate and succinate as metabolites produced by these bacteria, and administration of acetate and succinate increased mitochondrial respiration after 5 days of mechanical overload (MOV).

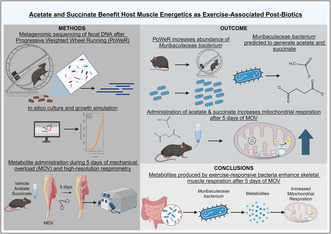

## 1 INTRODUCTION

The gut microbiome plays important roles in metabolism (Backhed et al., 2004, 2007). Exercise has been shown to induce composition and functional changes to the microbiome resulting in beneficial adaptations in the host (Allen et al., 2018; Kang et al., 2014; Monda et al., 2017). Microbial changes following exercise training appear to augment exercise capacity and improve host metabolism (Liu et al., 2020; Scheiman et al., 2019). There is also growing evidence that certain bacterial species may provide beneficial effects on skeletal muscle mass and function, and gut microbial metabolites link the microbiome to host physiology (Bindels et al., 2012; Chen et al., 2016; Krautkramer et al., 2021). In fact, roughly 10% of the metabolites in circulation are thought to be derived from the microbiome in mice (Wikoff et al., 2009), and over 833 gut microbial metabolites have been identified in humans (Han et al., 2021), representing a large reservoir of bioactive molecules.

Aoi and colleagues determined that transplanting the microbiome of exercise‐trained donor mice into germ‐free mice resulted in increased activation of AMP‐activated protein kinase (AMPK), calcium/calmodulin‐dependent protein kinase II (CaMKll), and Akt substrate of 160 kDa (AS160) in the gastrocnemius muscle (Aoi et al., 2023). Microbiota from exercise‐trained mice improved glucose tolerance after 8 weeks of high‐fat diet feeding, which was mediated by modification of circulating bile acids (Aoi et al., 2023). Additional studies have linked short chained fatty acids (SCFA) as gut microbial compounds that can regulate skeletal muscle mass (Chen et al., 2022; Han et al., 2022; Lahiri et al., 2019). Our laboratory, as well as others, recently showed that the gut microbiome is required for exercise‐induced muscle adaptations, including exercise capacity (Okamoto et al., 2019) and limb muscle hypertrophy (Valentino et al., 2021).

In this report, we performed an in‐depth bioinformatics analysis of the metagenomics data from microbiomes of sedentary and exercise‐trained mice. The goal of the current study was to determine how exercise training changes the composition and function of the gut microbiome, and whether exercise‐responsive bacterial metabolites can enhance muscle hypertrophy. To this end, we used our metagenomic data to perform computational simulations to identify key bacterial‐derived metabolites induced by progressive weighted wheel running (PoWeR). These metabolites were then administered to mice as post‐biotics during mechanical overload‐induced muscle hypertrophy. The findings of this study provide further evidence for a potential role of the gut microbiome in skeletal muscle adaptation to exercise training via microbial‐derived metabolites.

## 2 MATERIALS AND METHODS

### 2.1 Animals and ethical approval

Animal procedures were approved by the Institutional Animal Care and Use Committee at the University of Kentucky. All mice were housed in the same temperature‐ and humidity‐controlled room on a 14 h:10 h light–dark cycle. Mice were provided ad libitum access to food and water. At the end of the study, isoflurane (1%–2%) was administered, and once they did not respond to a firm pinch of the foot, mice were euthanized by exsanguination, followed by excision of the heart (to ensure death) under the administration of isoflurane. Some data reported in this study were generated using samples obtained from a previous study, which contains more detailed methods (Valentino et al., 2021). Briefly, all mice were adult (4 months of age) female C57BL/6J obtained from The Jackson Laboratory. Exercise training consisted of 8 weeks of progressive weighted wheel running (PoWeR) protocol, leading to a maximum of 6 g of added weight as previously described (Dungan et al., 2019). Mice were singly housed in cages with either a locked or unlocked running wheel. Both the sedentary and PoWeR groups were provided the same rodent chow (Teklad 2918 protein rodent diet; Envigo, Indianapolis IN) and water ad libitum. Feces was collected by placing a single mouse in a sterile plastic cage void of any bedding. Once the mouse had defecated, the feces was removed and stored at −80°C until further processing. For the pre‐exercise training timepoint, feces were collected immediately after a four‐week acclimation period, prior to the initiation of exercise. The post‐exercise feces samples were collected 24–48 h prior to euthanasia. Feces was collected by the same individual for each timepoint, roughly between 12 pm and 4 pm. Fecal DNA was isolated using a PureLink Microbiome DNA Purification Kit (Invitrogen; Catalog# A29790), and metagenomics sequencing of isolated fecal DNA was performed by CosmosID Inc. Fecal DNA concentration was quantified via Qubit (Thermo Fisher Scientific), and DNA libraries were prepared using IonExpress Plus Fragment kit (Thermo Fisher Scientific; Catalog# 4471269) according to the manufacturer's protocol. Library quantity was assessed with Qubit, and sequencing was performed on an Ion S5 XL sequencer (Thermo Fisher Scientific).

### 2.2 Species compositional analysis of metagenomics data

Metagenomic analyses were performed using the HUMAnN 3.0 pipeline (Beghini et al., 2021). Briefly, quality control for metagenomics sequences was performed using KneadData, which uses Trimmomatic (Bolger et al., 2014) for quality and adapter trimming and Bowtie2 (Langmead et al., 2019; Langmead & Salzberg, 2012) for mouse host and human contaminant sequence depletion. Processed reads were aligned to the ChocoPhlAn 3 database (mpa_v30_CHOCOPhlAn_201901) using MetaPhlAn 3 with the following options “‐‐add_viruses ‐‐ignore_eukaryotes –stat_q 0.01 ‐‐avoid_disqm.” The resulting relative abundance profiles for all samples were imported into R for subsequent analysis. The R package VEGAN v2.6–4 (Dixon, 2003) was used to calculate Shannon and Simpson indices.

### 2.3 Bacterial metabolite simulation

Bacterial genome for *Muribaculaceae bacterium DSM 103720* (PRJNA434628, GCA_003024805.1) was downloaded from the National Center for Biotechnology Information (NCBI). The genome assembly was analyzed using gapseq (Zimmermann et al., 2021) to predict reactions, pathways, and transporters, followed by model building and gapfilling as instructed by the program documentation. The model built from the bacterium genome was added to a virtual culture dish (20×20 grid) using a virtual growth medium. Simulation of bacterial growth was performed using BacArena (Bauer et al., 2017). Strongly changing metabolites were monitored for the duration of the simulation, and amounts (fmol) of SCFAs (butyrate, acetate, lactate), and succinate were plotted, and flux for each of these metabolites were calculated.

### 2.4 Mechanical overload induced muscle hypertrophy

Mice underwent bilateral sham or synergist ablation surgery, which mechanically overloads the plantaris muscle, as previously described by our group (Vechetti Jr. et al., 2021). Briefly, 4‐month‐old female C57BL/6J mice obtained from The Jackson Laboratory were anesthetized (isoflurane 1%–2%) and placed in prone position with both hindlimbs immobilized. Mice were operated on once they did not respond to a firm pinch of their foot. After hair removal, an incision on the skin from the mid‐belly of the gastrocnemius muscle down to the ankle was created to expose the Achille's tendon. The plantaris tendon was separated from the gastrocnemius and soleus tendons, and a rounded probe was used to separate the fascia between the plantaris muscle and the other synergist muscles. The soleus and roughly one third of the gastrocnemius muscle were excised. The excision was closed using Silk 5 sutures, and the plantaris was allowed to grow for 5 days. Mice were given vehicle (*n* = 6) (NaCl, 1.46 mg/mL) (Sigma Aldrich; Catalog# S9888), sodium acetate (*n* = 6) (1.97 mg/mL) (Okamoto et al., 2019) (Sigma Aldrich; Catalog# 241245), or sodium succinate (*n* = 6) (2.25 mg/mL) (Wang et al., 2020) (Sigma Aldrich; Catalog# 224731) through drinking water ad libitum. Vehicle was used to control for sodium intake.

### 2.5 Muscle histology and immunohistochemistry

Muscle cell size and fiber‐type distribution were assessed using previously described methodology (Dungan et al., 2019). Briefly, plantaris muscles after harvesting were embedded in Tissue TEK Optimal Cutting Temperature (O.C.T©, Sakura Finetek) and frozen using liquid nitrogen chilled isopentane, and 7 μm thick cryosections were immunolabeled using primary antibodies against Type IIA Myosin Heavy Chain (DSHB; Catalog# SC.71, Mouse IgG1) and laminin (Sigma Aldrich, Catalog# L9393, rabbit IgG). Fibers without Type IIA immunolabel were designated as Type IIB/X fibers. Type I fibers were not analyzed because they compose less than 1% of fibers in the mouse plantaris. Fiber‐type distribution and fiber cross‐sectional area were quantified using MyoVision2 (Viggars et al., 2022).

### 2.6 High‐resolution respirometry

After harvesting plantaris muscles and removal of visible fat and connective tissue, a portion of the sample (~10 mg wet weight) was immediately submerged in ice‐cold relaxation buffer (BIOPS; 10 mM Ca‐EGTA (Sigma Aldrich; Catalog# C4830), 0.1 μM free calcium, 20 mM imidazole (Sigma Aldrich; Catalog# 56750), 20 mM taurine (Sigma Aldrich; Catalog# T0625), 50 mM K‐MES (Sigma Aldrich; Catalog# M8250), 0.5 mM DTT (Sigma Aldrich; Catalog# D0632), 6.56 mM MgCl_2_ (Sigma Aldrich; Catalog# M9272), 5.77 mM ATP (Sigma Aldrich; Catalog# A2383), and 15 mM phosphocreatine (Sigma Aldrich; Catalog# P7936), pH 7.1). Respirometry was performed on only a subset of the samples (*n* = 6 vehicle, *n* = 4 acetate, and *n* = 4 succinate) analyzed by immunohistochemistry due to time constraints related to respirometry sample storage. Samples were kept on ice until time of preparation, and all steps of sample preparation were performed on ice. Muscle tissue was gently blotted with sterile gauze and then weighed using an analytical scale. Samples were moved to a glass vessel, and 100 μL respiration media (Mir05, 110 mM sucrose, 0.5 mM EGTA, 60 mM lactobionic acid, 3 mM MgCl_2_‐6H_2_O, 20 mM taurine, 10 mM KH_2_PO_4_, 20 mM HEPES, pH 7.1, 1 mg/mL BSA) (Oroboros Instruments, Innsbruck, Austria; Catalog# 60101–01) was added per mg of tissue. Samples were then disrupted by 6–8 strokes using a Teflon pestle powered by a Tri‐Rotir‐R (model K43) mixer (Tri‐R Instruments, Rockville Center) to obtain a tissue homogenate preparation, as previously described (Jiroutkova et al., 2015; Ziak et al., 2015). Mitochondrial respiration was then immediately measured with an Oroboros Oxygraph‐2 k (O2k) FluoRespirometer (Oroboros Instruments). Respiration media consisted of Mir05 buffer supplemented with 20 mM creatine monohydrate (Sigma Aldrich; Catalog# 27900). A substrate inhibitor titration (SUIT) protocol was performed to assess complex‐specific mitochondrial respiration. Pyruvate (5 mM) (Sigma Aldrich; Catalog# P2256) and malate (2 mM) (Sigma Aldrich; Catalog# M1000) were added to the oxygraph chambers to measure basal complex I, state 2 respiration. This was followed by ADP (4 mM) (Sigma Aldrich; Catalog# 117105) addition, to initiate state 3 (ADP‐stimulated) respiration. Succinate (10 mM) (Sigma Aldrich; Catalog# S2378) was added to stimulate electron flow through Complex II, followed by a titration of carbonyl cyanide m‐chlorophenyl hydrazine (CCCP, 0.25 μM to 1.5 μM) (Sigma Aldrich; Catalog# C2759) to stimulate maximal uncoupled respiration (*ET* capacity). Rotenone (10 μM) (Sigma Aldrich; Catalog# R8875) was used to inhibit complex I, and 5 μM antimycin A (Sigma Aldrich; Catalog# A8674) was used to inhibit electron flow through complex III, accounting for non‐mitochondrial oxygen consumption. Cytochrome c addition (10 μM) (Sigma Aldrich; Catalog# C7752) verified acceptable damage to the outer mitochondrial membrane, with increments of oxygen flux response after cytochrome c addition ranging from 10% to 15%. After completion of the SUIT protocol, samples were removed from the oxygraphy chambers, a DC protein assay kit (Bio‐Rad Laboratories, Hercules, CA; Catalog# 5000111) was used to measure total protein concentrations, and the respiration rate was normalized to protein content.

### 2.7 Citrate synthase activity assay

For estimation of mitochondrial content, the activity of citrate synthase (CS) was determined using an enzymatic assay. Lysed tissue homogenates were mixed in buffer containing Tris (100 mM, pH 8.0), 5,5′‐Dithiobis‐(2‐nitrobenzoic acid) (DTNB) (10 mM) (Sigma Aldrich; Catalog# D8130), acetyl CoA (30 mM) (Sigma Aldrich; Catalog# A2056), and oxaloacetic acid (OAA) (10 mM) (Sigma Aldrich; Catalog# O4126). CS activity was measured by monitoring the change in absorbance at 412 nm, which indicates the formation of 5‐thio‐2‐nitrobenzoic acid (TNB) upon the CS‐catalyzed reaction between acetyl CoA and DTNB in the presence of OAA. Absorbance was determined for 10 min using a Cytation 5 Multimode reader (Biotek, Winooski, VT), and CS activity was normalized to total protein content.

### 2.8 Western blotting

Total protein from lysed tissue homogenates was prepared in Laemmli sample buffer (Bio‐Rad; Catalog# 1610737), heated to 50°C for 10 min, and separated by SDS‐PAGE using 4%–15% Criterion TGX Precast Midi Protein Gels (Bio‐Rad; Catalog# 5671083). Proteins were then transferred by wet tank transfer for 1 h 30 min at constant current (0.2 A) onto a PVDF membrane (Bio‐Rad; Catalog# 1620239) in 20% methanol Tris‐glycine buffer (Bio‐Rad; Catalog# 1610771) at 4°C. Total protein was detected by Ponceau S staining (Thermo Fisher Scientific; Catalog# A40000279), and after washing with Tris‐buffered saline‐Tween (TBS‐T, 0.1% Tween‐20), membranes were blocked with SuperBlock Blocking Buffer (Thermo Fisher Scientific; Catalog# 37537) for 1 h at room temperature. Membranes were then incubated for 1 h 30 min at room temperature in OxPhos Rodent Antibody Cocktail (Thermo Fisher Scientific; Catalog# 45–8099) at a 1:1000 dilution, followed by washing in TBS‐T and incubation in goat anti‐mouse IgG, HRP‐conjugated secondary antibody (1:10,000) (Thermo Fisher Scientific; Catalog# 31430) for 1 h at room temperature. Blots were developed with Radiance ECL substrates (Azure Biosystems, Dublin, CA; Catalog# AC2204), imaged using a ChemiDoc MP system (Bio‐Rad), and quantified using Image Lab Software (Bio‐Rad). Mitochondrial content and mitochondrial protein abundance were measured in only a subset of samples (*n* = 3 vehicle, *n* = 3 acetate, and *n* = 3 succinate) due to limited tissue availability from the remaining samples.

### 2.9 Statistical analysis

A two‐way ANOVA with Sidak's multiple comparisons test was used to test differences in species, Shannon, and Simpson diversity. A two‐tailed Wilcoxon rank‐sum test was used to identify species that had significant (*adj. p* < 0.05) changes in relative abundances post‐ versus pre‐exercise, and false discovery rates were estimated using the Benjamini–Hochberg method. Co‐abundance network was calculated using Spearman correlation of species relative abundances and plotted using the igraph package (Csardi & Nepusz, 2006). Zero values were additively smoothed by the minimal non‐abundance among observed measurements before fold change calculations. Log transformed fold changes were used to calculate Bray distances for the non‐metric multidimensional scaling analysis using the “adonis2” function with 999 permutations.

For histology, mitochondrial respiration, citrate synthase activity, and western blot experiments, differences between acetate and succinate groups from vehicle were analyzed by a one‐way ANOVA, with Dunnett's method for multiple comparisons. The Shapiro–Wilk test was used to test the normality of the data. Analyses were performed in GraphPad Prism (v9.00, GraphPad Software), and statistical significance was set at an *α* < 0.05.

## 3 RESULTS

### 3.1 PoWeR induces modest change in gut microbiome diversity

**FIGURE 1:**
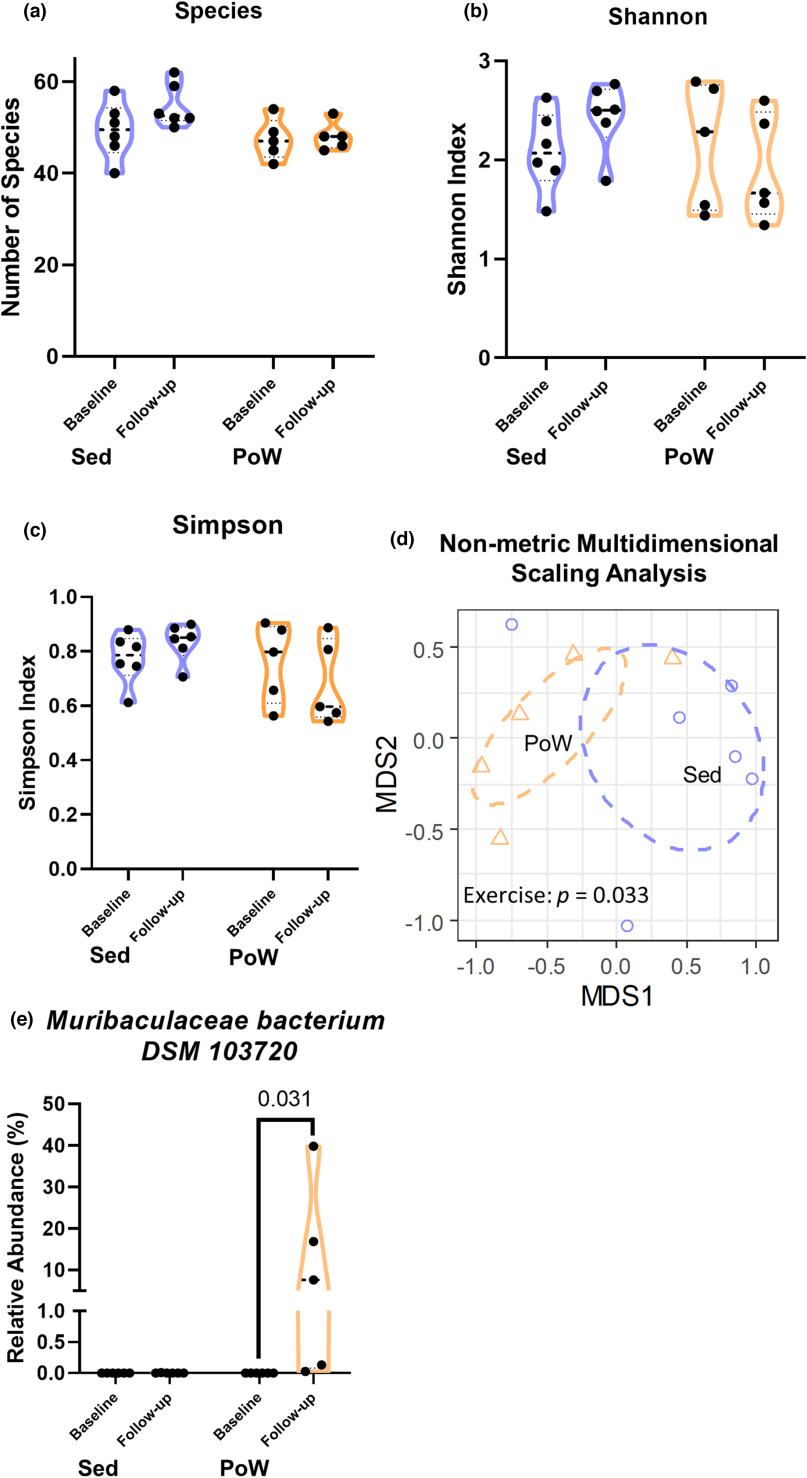
Exercise confers limited impact to the gut microbiome. Effect of exercise on microbiome biodiversity measures before (baseline) and after (follow‐up) 8‐weeks of PoWeR training (a) Number of microbial species, (b) Shannon index, (c) Simpson diversity index. Difference measured by a two‐way ANOVA with Sidak's multiple comparisons test. (d) Multidimensional scaling analysis (e) Exercise significantly increases the level of the bacterial species *Muribaculaceae bacterium DSM 103720*, assessed using a two‐tailed Wilcoxon rank‐sum test with Benjamini–Hochberg correction. *p*‐values shown for significant comparisons (adj. *p* < 0.05). PoW, exercise trained; Sed, sedentary; *n* = 5–6 mice per group.

We used PoWeR, a novel progressive endurance exercise training mouse model that lasted for a total of 8 weeks ending with 6 g of added weight to the running wheel (Valentino et al., 2021). Mice undergoing PoWeR training were a subset of mice from a previous study in which the average weekly volume was 12.71 ± 1.15 km/wk. There were no statistically significant differences in the number of bacterial species after exercise (Figure 1a). Similarly, there was no significant difference in alpha diversity, assessed by the Shannon index, which accounts for both the richness and evenness of species in a community (Figure 1b). Furthermore, there was no differences in the Simpson index, which takes into account both the number of species and the abundance of those species, following exercise training (Figure 1c). Non‐metric multidimensional scaling analysis (Figure 1d) demonstrated a significant (*p =* 0.033) effect of exercise on the β‐diversity (a measure of the different microbial communities between groups) between the sedentary and PoWeR‐trained mice (Figure 1d). These results suggest that PoWeR training induces moderate changes to the composition of the microbiome without affecting species richness and evenness. With these results, we next aimed to determine whether there were species unique to the microbiome of the PoWeR‐trained group compared to the sedentary group.

### 3.2 PoWeR induces higher abundance of Muribaculaceae bacterium DSM 103720

Our initial analysis of the microbiomes between sedentary and PoWeR‐trained mice revealed that exercise caused a significant change in β‐diversity. Thus, we set out to identify candidate bacterial species that respond to exercise. We employed a paired study design comparing pre‐ to post‐sedentary and PoWeR groups; importantly, mice in the sedentary group were singly housed in locked wheel cages to control for the potential impact of the new environment on the gut microbiome. We identified bacterial species whose abundance significantly changed in the sedentary and PoWeR‐trained groups when comparing pre‐ vs post‐relative species abundances. Although there were multiple bacterial species that showed significant increases in abundance in response to PoWeR, only one species, *Muribaculaceae bacterium DSM 103720* (Figure 1e, adj. *p* = 0.031), was unique to the PoWeR group while other species also showed increases in sedentary controls. Figure S1 shows changes in species abundances for sedentary and PoWeR‐trained mice. A majority of species decreased after exercise, although changes were not statistically significant. Data in Figure S1 are also provided in Table S1.

### 3.3 Co‐abundance network analysis identifies potentially important secondary bacterial species

**FIGURE 2:**
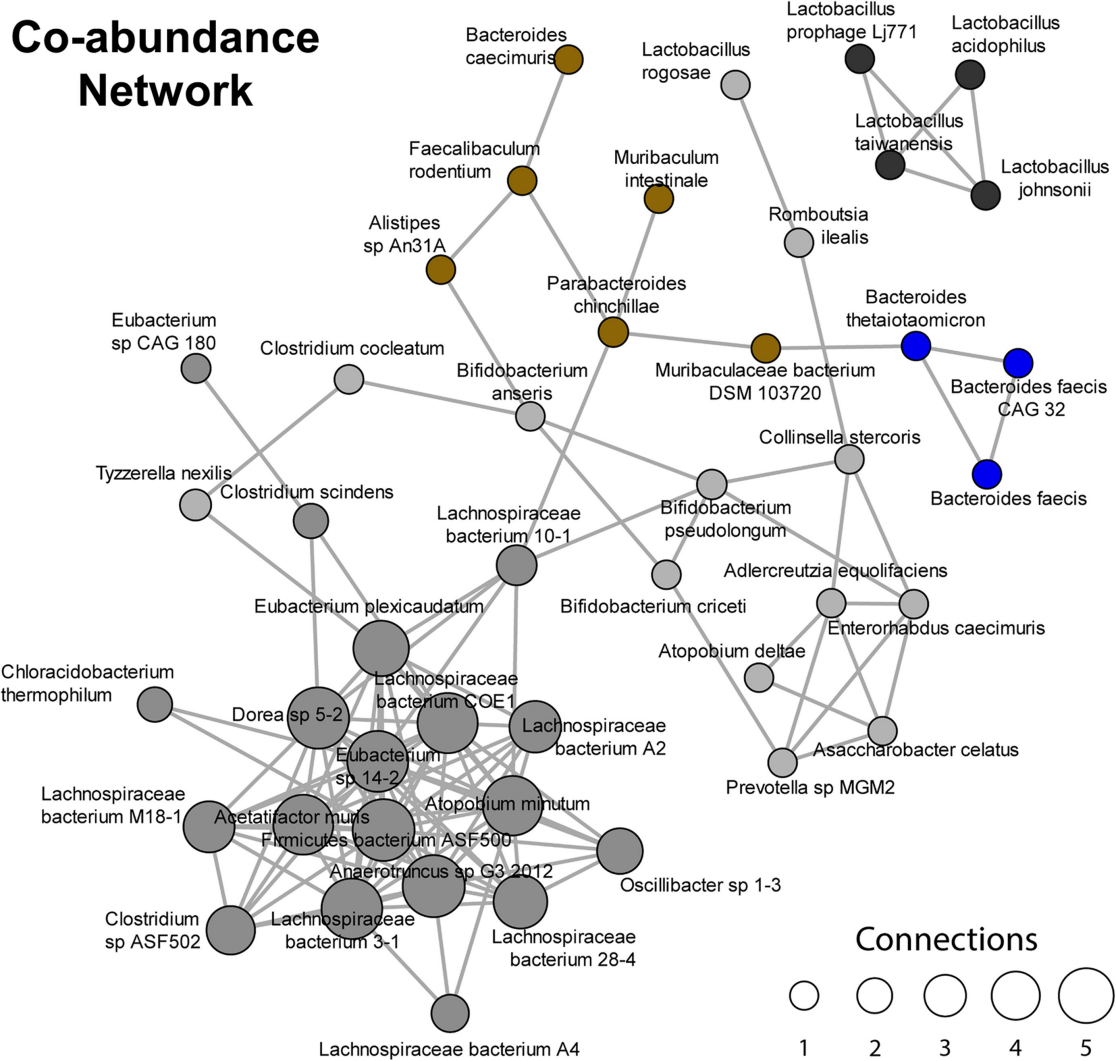
Co‐abundance network analysis among the gut microbiome after period of exercise. A graphical microbial interaction network capturing species that change in relative abundance in a coordinated manner. Each node corresponds to microbial species, and each edge represents a significant Spearman correlation (either positive or negative). Colors are selected arbitrarily but indicate clustering of species with similar correlations, suggesting cross‐feeding relationship or co‐dependence.

We next used co‐abundance network analysis to identify other bacterial species that shared a similar pattern of expression as *Muribaculaceae bacterium* (Figure 2). This analysis shows significant correlations, both positive and negative, as connections in a network graph, and highlights species that are codependent in the microbiome. The connections and hubs outline the community structure of all the species in the microbiome and provide insight into the levels of closeness and separation among species. *Muribaculaceae bacterium DSM 103720* was part of a small cluster of species that included five other species (brown cluster), with *Parabacteroides chinchillae and Faecalibaculum rodentium* being at the center of the cluster. Additionally, *Muribaculaceae bacterium DSM 103720* was negatively associated with *Bacteroides thetaiotamicron*, which was part of a cluster with two other species that were the most decreased after exercise (blue cluster).

### 3.4 Simulations identify acetate and succinate as predominant microbial‐derived metabolites

**FIGURE 3:**
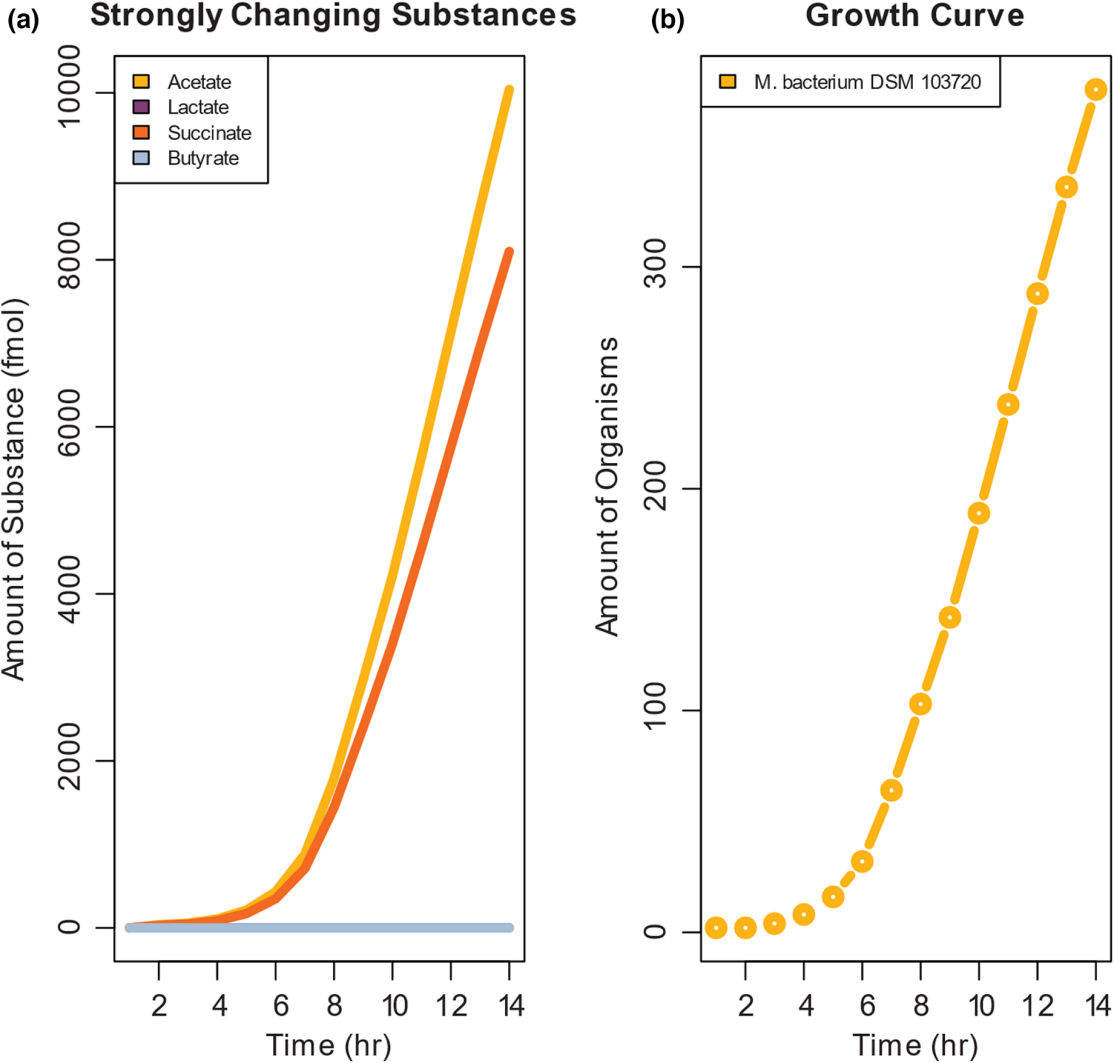
In silico growth and metabolite simulations predict acetate, succinate, and butyrate as major exercise‐responsive bacterial metabolites. Acetate and succinate are two metabolites that accumulate at the highest rate in growth medium (a) during simulated culture (b) of *Muribaculaceae bacterium DSM 103720*. All bacteria models were constructed by gapseq (Zimmermann et al., 2021) and simulated using BacArena (Bauer et al., 2017).

Having identified species whose abundance changed in response to PoWeR, we next wanted to identify potential microbial‐derived metabolites that might contribute to muscle adaptation. We employed a computational prediction tool, gapseq (Zimmermann et al., 2021), that analyzes bacterial genomes for enzymes and overrepresented pathways, and performs in silico culture and growth simulations along with metabolites generated in the “growth media.” Using gapseq, we simulated the growth and interactions of *Muribaculaceae bacterium DSM 103720*. We found that acetate and succinate were the two most robustly synthesized metabolites in the simulated growth of *Muribaculaceae bacterium DMS 103720* (Figure 3a). *Muribaculaceae bacterium DMS 103720* had a high growth rate that did not plateau by the end of simulated growth time (Figure 3b).

### 3.5 Succinate and acetate do not enhance muscle growth but increase mitochondrial respiration following 5 days of MOV‐induced muscle hypertrophy

**FIGURE 4:**
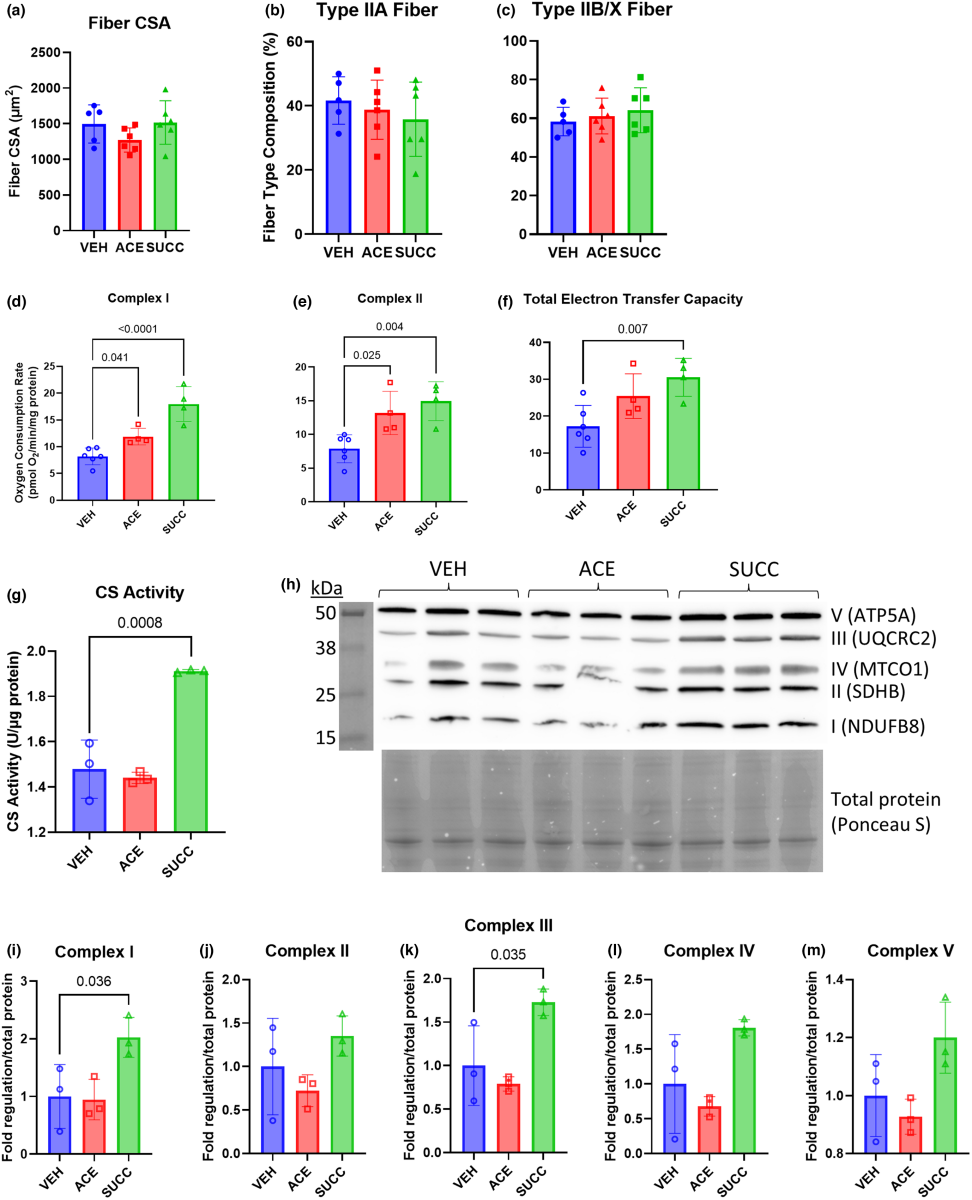
Succinate and acetate supplementation increase mitochondrial respiration following 5 days of mechanical overload (MOV). (a) Mean fiber cross‐sectional area (CSA) after 5 days of MOV with acetate (ACE) or succinate (SUCC) compared to vehicle (VEH) administration, (b) Type IIA, and (c) Type IIB/X fiber‐type composition. Mitochondrial respiration in plantaris tissue homogenates after 5 days of MOV (d–f). Complex I oxygen consumption rate (d), complex II oxygen consumption rate (e), and electron transfer capacity (f) after 5 days of MOV, normalized to total protein content. *n* = 4–6 animals per group. (g) Citrate synthase (CS) enzymatic activity and (h) Western blotting of oxidative phosphorylation (OxPhos) subunit proteins in plantaris tissue homogenate lysates following 5 days of MOV. Electron transport chain complex and subunit shown on right side, molecular weight ladder shown on left side. OxPhos protein levels normalized to total protein, determined by Ponceau S staining (shown below). (i–m) Quantification of the fold change of protein levels of subunits of (i) complex I, (j) complex II, (k) complex III, (l) complex IV, and (m) complex V. NDUFB8: NADH Ubiquinone Oxidoreductase Subunit B8, SDHB: Succinate Dehydrogenase Complex Iron Sulfur Subunit B, MTCO1: Mitochondrially Encoded Cytochrome C Oxidase I, UQCRC2: Ubiquinol‐Cytochrome C Reductase Core Protein 2, ATP5A: ATP synthase F1 subunit alpha. Bars are Mean ± SD. Differences relative to VEH tested using one‐way ANOVA, with Dunnett's method for multiple comparisons. *P*‐values shown for significant comparisons (*p* < 0.05).

PoWeR is a unique exercise model of progressive endurance exercise training, inducing hypertrophy, myonuclear accretion, and fiber‐type shift adaptations (Dungan et al., 2019). The results of the in silico culture simulation indicate that PoWeR‐responsive *Muribaculaceae bacterium DSM* 103720 is capable of producing high levels of acetate and succinate. Although previous studies have assessed the effects of short‐chain fatty acids such as acetate and succinate administration on endurance training performance (Carey & Montag, 2021; Pan et al., 2015; Seike et al., 2020; Wang et al., 2020; Xu et al., 2021), how these metabolites might impact hypertrophic growth is not known. Thus, we sought to investigate the effect of acetate or succinate supplementation on mechanical overload (MOV)‐induced muscle hypertrophy. We administered acetate or succinate to mice in their drinking water while controlling for sodium intake, then subjected the plantaris muscles to 5 days of MOV. There was no significant difference in fiber cross‐sectional area (Figure 4a) or fiber‐type composition (Figures 4b,c) with either acetate or succinate administration compared to vehicle.

Despite a lack of impact on muscle size, succinate and acetate treatment may affect other aspects of muscle physiology during MOV. Exogenous treatment with succinate has been previously shown to increase both oxygen consumption and the expression of genes related to the electron transport chain in skeletal muscle (Wang et al., 2020). Acetate treatment was also shown to enhance endurance capacity in mice where it could serve as a potential fuel source (Bertocci et al., 1997). Additionally, acetate treatment increased overall succinate dehydrogenase staining in rat soleus and gastrocnemius muscle, suggesting increased oxidative capacity (Maruta et al., 2022). MOV induces a heavy demand on protein synthesis in favor of building sarcomeric and cytoskeletal proteins, and evidence suggests that this may be at the expense of mitochondrial protein abundance (Uemichi et al., 2021). Thus, we sought to measure mitochondrial respiration in plantaris muscles of mice that underwent 5 days of MOV. We found that relative to vehicle‐treated mice, acetate and succinate led to elevated complex I (Figure 4d, *p* = 0.041 and *p* < 0.0001, respectively) and complex II respiration (Figure 4e, *p* = 0.019 and *p* = 0.003, respectively). Furthermore, succinate treatment significantly increased maximal uncoupled respiration (*p* = 0.007) (Figure 4f).

Succinate treatment also significantly increased CS activity in plantaris muscles following 5 days of MOV (*p* < 0.001) while acetate had no significant effect on CS activity. Similarly, western blot analysis of the relative levels of the 5 oxidative phosphorylation (OxPhos) complexes (Figure 4h) demonstrated that acetate had no effect on protein levels of any of the OxPhos subunits assessed (Figures 4i–m). However, succinate treatment significantly increased levels of complex I and complex III subunits relative to vehicle treatment (*p* = 0.046 and *p* = 0.035, respectively) (Figures 4i,k). Succinate treatment did not significantly affect protein levels of complex II, complex IV, or complex V subunits (Figure 4j,l,m).

## 4 DISCUSSION

Exercise is known to modulate the composition and function of the gut microbiome, but the modality of exercise, be it resistance or endurance, may mediate functional changes in the microbiome in slightly different ways. As previously demonstrated by Allen and colleagues, forced versus voluntary exercise also distinctly modulates the bacterial composition in mice (Allen et al., 2015). While little is known about the effect of strength or resistance training on the microbiome, here, we have presented data demonstrating that 8 weeks of PoWeR, a voluntary progressive endurance exercise training modality, caused a significant increase in a novel species, *Muribaculaceae bacterium DSM 103720*. Additionally, we found that PoWeR caused the co‐occurrence of distinct microbes, suggesting the importance of interspecies networking. Furthermore, our in silico simulations identified acetate and succinate as bacterial‐derived metabolites that may have a role in skeletal muscle adaptation to PoWeR training.

PoWeR training resulted in no changes to alpha diversity, a common metric for species diversity and evenness. This finding agrees with other studies showing either no change or a loss in alpha diversity in response to exercise (Allen et al., 2015; Craven et al., 2022; Zhong et al., 2021). The Simpson diversity index, which takes into account the abundance of each species, was also unchanged after PoWeR training. The microbial strain that was highly responsive to PoWeR training, *Muribaculaceae bacterium DMS 103720*, is a member of the family *Bacteroidales* (originally noted as *S24‐7*), which have the capacity to produce succinate, acetate, and propionate through fermentation of carbohydrates (Lagkouvardos et al., 2019; Ormerod et al., 2016). Previous studies have also found that voluntary wheel running results in a significant increase in *Muribaculaceae (*Lamoureux et al., 2017; Williams et al., 2023 *)*. In these studies, as well as the present study, mice ran on average 7–13 km/day. In contrast, during forced wheel running where mice trained for 40–60 min per training session, *Muribaculaceae* was not detected (Kang et al., 2014; Schonke et al., 2023). This suggests that at least one factor modulating the increase in *Muribaculacea* may be exercise volume. In support of this, Torquati et al. determined that training intensity and volume exert differences in microbial composition and functional changes in people with type 2 diabetes (Torquati et al., 2023). Notably, our metagenomic analysis did reveal a high variability in the abundance of *Muribaculaceae bacterium DMS 103720* post‐PoWeR training, which may be related to the variability in running volume. Another potential factor is the responders vs. non‐responders effect in microbial changes, as observed by Lui and colleagues (Liu et al., 2020). As with other physiological variables with responders/non‐responders, the microbiome may also demonstrate inconsistent adaptation between samples (Diener et al., 2021). The resistance of a microbial community to change under a given stimulus could indicate the variability in microbiome stability between samples.

The co‐abundance network analysis revealed species of bacteria that may be strongly associated with changes in the abundance of *Muribaculaceae bacterium DMS 103720*. Thus, the exercise‐responsive microbes may need other species to facilitate their production of metabolites. Recently, the microbiome has been described as a functionally cohesive unit rather than a collection of independent species with microbes producing metabolites required by their neighbors (Ghanbari Maman et al., 2020). Daisley and colleagues highlight this paradigm shift through what they term the “pantryome,” which emphasizes how microbes have common bioenergetic machinery to support their functional outcomes (Daisley et al., 2021). The significant increase in *Muribaculaceae bacterium DMS 103720* was also negatively correlated with the most significantly decreased species, *Bacteroides thetaiotamicron*. The loss of certain species may also be beneficial for muscle adaptation to exercise whether those species were producing metabolites that have a detrimental effect or compete with the growth of positive exercise‐responsive microbes. The significance of this finding requires further investigation.

The in silico simulation predicts that acetate and succinate are mainly produced by *Muribaculaceae bacterium DMS 103720*. Acetate, a two‐carbon short‐chain fatty acid (SCFA), is a major microbial produced metabolite. Microbial‐derived SCFAs, including acetate, have been shown to benefit multiple host organs (Perry et al., 2016; Zhao et al., 2020; Zheng et al., 2021), including muscle mass and function (Lahiri et al., 2019; Sakakida et al., 2022). Acetate also improves exercise capacity (Okamoto et al., 2019). Likewise, Reddy et al. recently determined that succinate contributed to endurance exercise‐induced muscle adaptations (Reddy et al., 2020). Dietary supplementation of succinic acid also has been shown to lead to enhanced endurance capacity, muscle grip strength, and fast‐ to slow‐twitch fiber transition in mice via Succinate receptor 1 (SUCNR1) signaling (Wang et al., 2020).

While endurance and resistance exercise elicit certain shared training responses, they are also characterized by several distinct adaptations and mechanisms, including unique secretomes (Leuchtmann et al., 2021). PoWeR is characterized by a progressive endurance training‐like stimulus, where both endurance and resistance adaptations occur (Dungan et al., 2022). Resistance training‐induced hypertrophy, mediated largely by mechanical activation, can be modeled by synergist ablation‐induced MOV, which promotes robust muscle hypertrophy (Kirby et al., 2016). Our laboratory has shown significant increases in total RNA content as well as plantaris muscle wet weight as early as 3–5 days after MOV (Kirby et al., 2016; Miyazaki et al., 2011). In the current study, we found that acetate and succinate did not result in enhanced hypertrophy after 5 days of MOV. While this does not rule out the importance of these metabolites in skeletal muscle growth, they do not appear to effect hypertrophy during the first 5 days of MOV. Future research is needed to understand whether acetate and succinate support skeletal muscle hypertrophy at longer timepoints. Moreover, since early‐phase plantaris hypertrophy occurs very rapidly with synergist ablation (Roberts et al., 2020), studies should evaluate acetate and succinate in models that allow for slower, progressive hypertrophy. Furthermore, in addition to muscle size, future research should assess the effects of acetate and succinate on muscle strength.

MOV‐induced skeletal muscle hypertrophy is driven by increases in protein synthesis, a bioenergetically costly process that consumes up to 30% of the total ATP pool (Sartori et al., 2021). Acetate and succinate can participate in energy producing pathways (Martinez‐Reyes & Chandel, 2020), which may support the energy demanding pathways initiated in the acute days following MOV. Notably, previous transcriptomic analyses of the acute phase of muscle mechanical loading induced by synergist ablation have demonstrated a downregulation of oxidative metabolism‐related gene expression (Chaillou et al., 2013; Murach et al., 2022). Likewise, Uemichi and colleagues reported a decline in *Pgc‐1α* expression, a key regulator of mitochondrial biogenesis, in plantaris muscles 14 days following synergist ablation (Uemichi et al., 2021). Protein levels of the oxidative phosphorylation proteins succinate dehydrogenase (SDHB, complex II), cytochrome b‐c1 complex subunit 2 (UQCRC2, complex III), and ATP synthase F1 subunit alpha (ATP5A, complex V) were also all significantly reduced in response to MOV (Uemichi et al., 2021). The existence of competition between mitochondrial and ribosome biogenesis, with a prioritization of the latter during MOV‐induced hypertrophy, has been proposed as a potential cause of lower oxidative metabolism during rapid MOV‐induced hypertrophy (Mesquita et al., 2021). Thus, modulation of the cellular energy state under the metabolically stressed environment of MOV may be useful to match the heightened ATP demand in the hypertrophic adaptive process.

Succinate, an intermediate of the tricarboxylic acid (TCA) cycle, directly interacts with the mitochondrial electron transport chain via complex II, enabling a “shortcut” route to ATP production (Protti, 2018). Previous reports in glial cells and fibroblasts (Bakare et al., 2021; Giorgi‐Coll et al., 2017), as well as in skeletal muscle (Xu et al., 2021), have demonstrated that succinate can increase oxidative phosphorylation. In our functional analyses of the oxygen consumption rate of mechanically loaded muscles, we found complex II respiration increased with succinate supplementation compared to vehicle. Interestingly, succinate also led to increased complex I respiration. Thus succinate, which bypasses complex I, may lead to increased ATP production and maintained membrane potential, which can reduce metabolic demands on complex I (Ehinger et al., 2016). Importantly, complex I is especially vulnerable to ROS, which can be induced by MOV (Ehinger et al., 2016). The increase in mitochondrial respiration with succinate administration may have been mediated by the increased mitochondrial content and OxPhos protein levels observed in the overloaded plantaris muscles of succinate‐treated mice. Notably, succinate has been shown to increase mitochondrial biogenesis and mitochondrial content in muscle (Wang et al., 2020).

In addition to succinate, acetate supplementation also led to elevated complex I and complex II respiration following MOV relative to vehicle treatment. Interestingly, however, the mechanism by which acetate improves mitochondrial respiration may not be related to increased mitochondrial biogenesis, as we found no difference in mitochondrial content or OxPhos protein levels in muscles of acetate‐treated mice compared to vehicle. Instead, the effects of acetate on mitochondrial function are thought to involve maintenance of acetyl‐CoA pools (Hu et al., 2020; Liu et al., 2018; Sahuri‐Arisoylu et al., 2016). Infusion of acetate has been demonstrated to ameliorate impairments in endurance exercise performance in antibiotic‐treated mice (Okamoto et al., 2019). Acetate supplementation also led to improved skeletal muscle post‐exercise glucose utilization in horses (Waller et al., 2009). The importance of acetate in muscle mitochondrial function is further exemplified by the high expression of the mitochondrial acetyl‐CoA synthetase enzyme *ACSS1* in skeletal muscle tissue, which catalyzes the synthesis of acetyl‐CoA from acetate (Moffett et al., 2020). The metabolism of acetate has been described as an important parallel pathway for acetyl‐CoA production during conditions of cellular stress, and acetate supplementation may provide an alternate fuel source for aerobic energy metabolism in the cellular environment during hypertrophic growth of muscle fibers induced by MOV as well (Liu et al., 2018).

Taken together, these data suggest that acetate and succinate may be useful metabolites for modulating oxidative metabolism during the physiological conditions associated with MOV. It should be noted that a limitation of our study is the sample group sizes in some of the experiments. Future research in a larger cohort will be necessary to further elucidate the utility of acetate and succinate as exercise‐associated post‐biotics and further clarify their mechanisms of action.

## AUTHOR CONTRIBUTIONS

AI: performed experiments, analyzed data, interpreted results of experiments, prepared figures, drafted manuscript, approved final version of manuscript. TV: conceived and designed research, performed experiments, analyzed data, interpreted results of experiments, drafted manuscript, approved final version of manuscript. BB: performed experiments, analyzed data, interpreted results of experiments, prepared figures, edited and revised manuscript, approved final version of manuscript. JG: performed experiments, edited and revised manuscript, approved final version of manuscript. TPS: performed experiments, edited and revised manuscript, approved final version of manuscript. FA: performed experiments, edited and revised manuscript, approved final version of manuscript. AO: conceived and designed research, interpreted results of experiments, edited and revised manuscript, approved final version of manuscript. JJM: conceived and designed research, analyzed data, interpreted results of experiments, edited and revised manuscript, approved final version of manuscript. YW: conceived and designed research, performed experiments, analyzed data, interpreted results of experiments, prepared figures, drafted manuscript, edited and revised manuscript, approved final version of manuscript.

## FUNDING INFORMATION

National Institute on Aging, National Institutes of Health, Grant Number: 5R21AG071888 (to JJM). National Institute of Arthritis and Musculoskeletal and Skin Diseases, National Institutes of Health, Grant Number: 5K99AR081367 (to YW). Intramural research program of the U.S. Department of Agriculture, National Institute of Food and Agriculture, [Agriculture and Food Research Initiative, grant no. 2022–67,012‐38,533, project accession no. 1029340] (to AI). The findings and conclusions in this publication have not been formally disseminated by the U.S. Department of Agriculture and should not be construed to represent any agency determination or policy.

## CONFLICT OF INTEREST STATEMENT

The authors declare that they have no competing interests.

## ETHICS STATEMENT

Animal procedures were approved by the Institutional Animal Care and Use Committee at the University of Kentucky.

## Supporting information


Figure S1.

Table S1.

## Data Availability

The datasets used and/or analyzed during the current study are available from the corresponding author on reasonable request.
