# Supplementary figures and images for: Acetate and succinate benefit host muscle energetics as exercise‐associated post‐biotics
Source: Physiol Rep. 2023 Nov 8;11(21):e15848. doi: 10.14814/phy2.15848 (PMC10632089; doi:10.14814/phy2.15848)

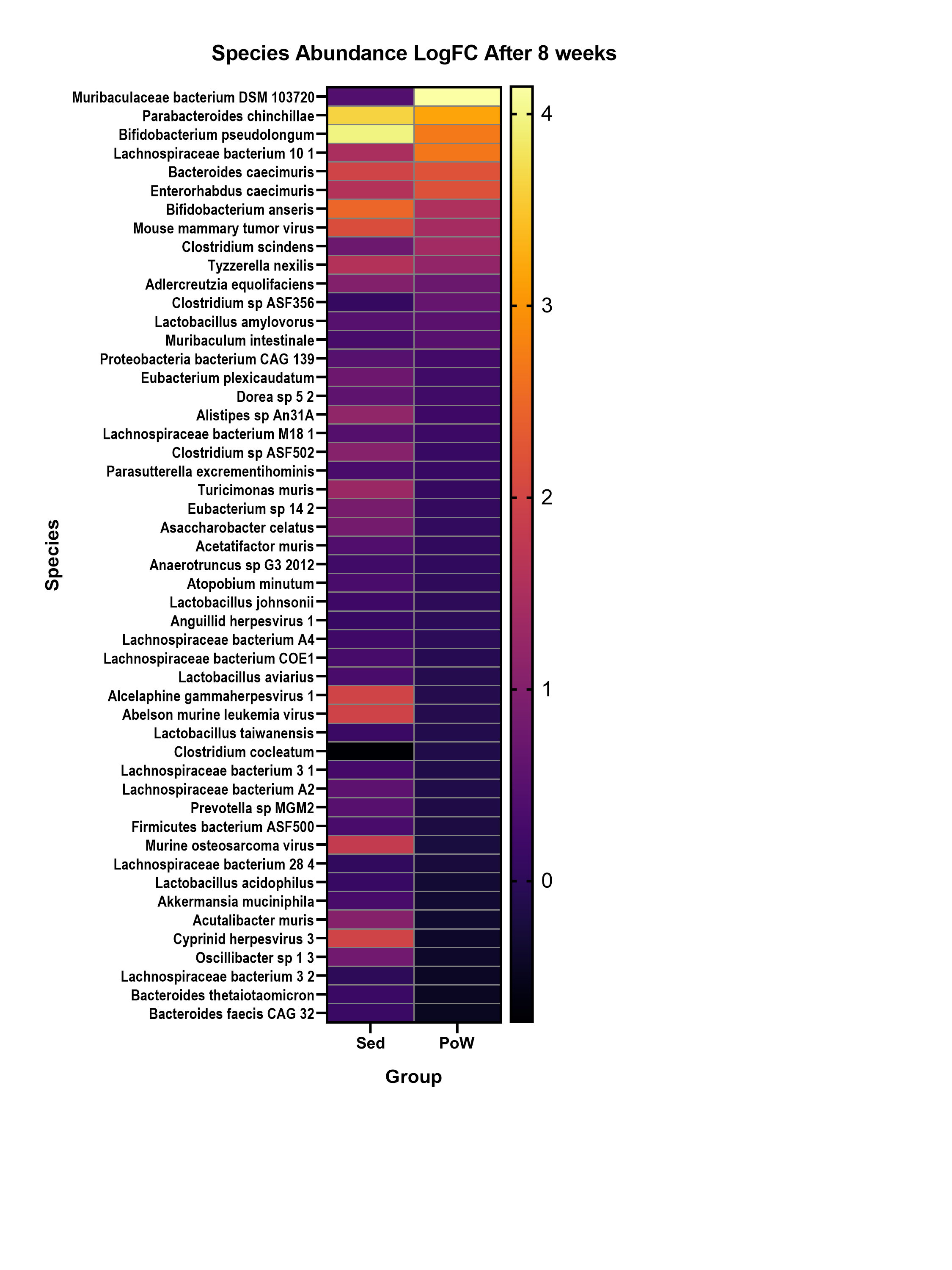

Supplement: Supplementary file 1 — Figure S1. [file PHY2-11-e15848-s001.tif]
